# First observation of tropospheric nitrogen dioxide from the Environmental Trace Gases Monitoring Instrument onboard the GaoFen-5 satellite

**DOI:** 10.1038/s41377-020-0306-z

**Published:** 2020-04-20

**Authors:** Chengxin Zhang, Cheng Liu, Ka Lok Chan, Qihou Hu, Haoran Liu, Bo Li, Chengzhi Xing, Wei Tan, Haijin Zhou, Fuqi Si, Jianguo Liu

**Affiliations:** 1 School of Earth and Space Sciences, University of Science and Technology of China, Hefei, 230026 China; 2 Department of Precision Machinery and Precision Instrumentation, University of Science and Technology of China, Hefei, 230026 China; 3 Key Laboratory of Environmental Optics and Technology, Anhui Institute of Optics and Fine Mechanics, Chinese Academy of Sciences, Hefei, 230031 China; 4 Center for Excellence in Regional Atmospheric Environment, Institute of Urban Environment, Chinese Academy of Sciences, Xiamen, 361021 China; 5 Key Laboratory of Precision Scientific Instrumentation of Anhui Higher Education Institutes, University of Science and Technology of China, Hefei, 230026 China; 6 Remote Sensing Technology Institute (IMF), German Aerospace Center (DLR), Oberpfaffenhofen, Germany

**Keywords:** Atmospheric optics, Optical spectroscopy

## Abstract

The Environmental Trace Gases Monitoring Instrument (EMI) is the first Chinese satellite-borne UV–Vis spectrometer aiming to measure the distribution of atmospheric trace gases on a global scale. The EMI instrument onboard the GaoFen-5 satellite was launched on 9 May 2018. In this paper, we present the tropospheric nitrogen dioxide (NO_2_) vertical column density (VCD) retrieval algorithm dedicated to EMI measurement. We report the first successful retrieval of tropospheric NO_2_ VCD from the EMI instrument. Our retrieval improved the original EMI NO_2_ prototype algorithm by modifying the settings of the spectral fit and air mass factor calculations to account for the on-orbit instrumental performance changes. The retrieved EMI NO_2_ VCDs generally show good spatiotemporal agreement with the satellite-borne Ozone Monitoring Instrument and TROPOspheric Monitoring Instrument (correlation coefficient *R* of ~0.9, bias < 50%). A comparison with ground-based MAX-DOAS (Multi-Axis Differential Optical Absorption Spectroscopy) observations also shows good correlation with an *R* of 0.82. The results indicate that the EMI NO_2_ retrieval algorithm derives reliable and precise results, and this algorithm can feasibly produce stable operational products that can contribute to global air pollution monitoring.

## Remote sensing: Tuned-up satellite identifies pollutant hot spots

Improvements to a high-resolution spectrometer onboard China’s recently-launched GaoFen-5 (GF-5) satellite can give researchers a new tool to assess atmospheric trace gases. Cheng Liu from the University of Science and Technology of China and his colleagues reported the first successful retrieval of nitrogen dioxide from the GF-5 orbiter thanks to crucial adjustments to calibration algorithms. The satellite’s payload Environmental Trace Gases Monitoring Instrument (EMI) uses visible spectroscopic measurements to detect nitrogen dioxide in the atmosphere, which can be used to identify the spatial distribution of pollution emissions on the Earth. To overcome on-orbit issues including detector saturation, the team has optimized the spectral retrieval settings by spectral precalibration, fitting wavelength adjustment, reference spectrum selection, etc. Comparisons to other satellite and ground measurements revealed the optimization effort yielded reliable results for monitoring pollution levels.

## Introduction

The Environmental Trace Gases Monitoring Instrument (EMI)^1^ is the first Chinese satellite-borne spectrometer with the aim to measure atmospheric pollutants from space. The EMI payload onboard the GaoFen-5 satellite was successfully launched on 9 May 2018. The GaoFen-5 satellite has a polar orbit at an altitude of 706 km. The Chinese EMI instrument is expected to contribute to the understanding of global air quality and atmospheric chemistry, similar to predecessor European and American satellite missions, e.g., the Ozone Monitoring Instrument (OMI)^2^ and TROPOspheric Monitoring Instrument (TROPOMI)^3^. EMI has instrumental characteristics that are similar to OMI and TROPOMI, e.g., the local overpass time at ~13:30, spectral coverage, push-broom imaging technique, and daily global coverage. Both EMI and TROPOMI (launched in 2017) are new-generation satellite-borne air pollutant sensors compared to the OMI that was launched in 2004. TROPOMI follows the heritage of OMI in both instrument design and trace gas retrievals, but with higher spatial resolution and signal-to-noise ratio. A prototype EMI nitrogen dioxide (NO_2_) retrieval algorithm was developed before launch based on the OMI NO_2_ retrieval. However, optimization of the NO_2_ retrieval algorithm was necessary to adapt the unexpected issues of EMI after launch, especially spectral calibration.

Nitrogen oxides (NO_x_), defined as the sum of nitrogen oxide and NO_2_, are the major pollutants contributing to ozone and secondary aerosol formation in the troposphere through photochemical reactions^4^. Sources of NO_x_ include fossil fuel combustion, vehicle emissions, biomass burning, and lightning^5^. Due to rapid industrialization and urbanization in the past few decades, China has become one of the largest NO_x_ emitters in the world^6^. As a result, China is experiencing a series of severe air pollution problems^7,8^. In addition to measuring NO_2_ distribution directly from space, applications of satellite remote sensing may include estimations of pollutant emissions^9^, air quality trend detection^10^, model validation, and assimilation of satellite data^11^.

**Fig. 1:**
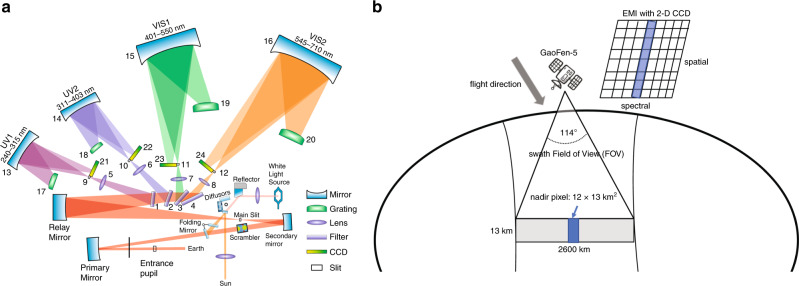
Illustration of the operating principle of the EMI instrument. **a** Schematic diagram of the EMI optical system. **b** The on-orbit operation of the EMI instrument onboard the GaoFen-5 satellite

Figure 1a illustrates the optical design of the EMI satellite instrument. The EMI instrument covers the ultraviolet (UV) and visible (Vis) spectral ranges from 240 to 710 nm with a spectral resolution of 0.3–0.5 nm. Light received by the telescope is depolarized by a scrambler and subsequently split into four spectral channels, the UV1 (240–315 nm), UV2 (311–403 nm), VIS1 (401–550 nm), and VIS2 (545–710 nm) channels. Each spectrometer is equipped with a two-dimensional charge-coupled device (CCD) detector, with one dimension used for spectral coverage and the other dimension used for spatial coverage. The EMI instrument scans in the nadir direction toward the earth’s surface with an opening angle of 114° corresponding to a swath width of 2600 km, enabling daily global coverage with a nadir resolution of 12 × 13 km^2^ and a local overpass time of 13:30 (Fig. 1b). The direct sun solar irradiance spectrum, typically used as a reference spectrum in the spectral analysis of the nadir radiance measurement, is introduced to the EMI telescope once a day using the quartz volume diffuser^12^. By using the unique absorption features of different trace gases in the UV–Vis range, the abundances of atmospheric pollutants can be retrieved from the difference between atmospheric and solar spectra.

In this paper, we present a new tropospheric NO_2_ vertical column density (VCD, i.e., the vertical integral of NO_2_ concentration from the earth’s surface to the top of the atmosphere) retrieval algorithm dedicated to the EMI instrument. Details of the spectral retrieval, stratospheric–tropospheric separation of the NO_2_ column and slant to vertical column conversion are presented. The first EMI retrieval of tropospheric NO_2_ columns is compared to datasets from modern state-of-the-art European and American satellite sensors.

## Results

### NO_2_ retrieval overview

The retrieval of tropospheric NO_2_ VCDs from satellite UV–Vis observations typically follows a state-of-the-art three-step approach. First, the total NO_2_ slant column density (SCD) are retrieved from nadir radiance spectra normalized by the solar irradiance, using the differential optical absorption spectroscopy (DOAS) technique^13^. Subsequently, the stratospheric NO_2_ columns are separated from the total NO_2_ SCDs by assuming longitudinal homogeneity of stratospheric NO_2_, while neglecting the minor contribution of tropospheric NO_2_ (usually on the order of 10^14^ molecules cm^−2^) over remote clean regions^14,15^. Last, tropospheric NO_2_ SCDs are converted to VCDs using air mass factors (AMFs)^16^. The AMF is defined as the ratio between SCD and VCD. It is a measure of the effective optical path length from the top of the atmosphere to the earth’s surface and reflected to the satellite through the atmosphere:

1 $$M = \frac{S}{V}$$

where *M* is the AMF, *S* denotes the SCD, and *V* represents the VCD. The AMF can be calculated with a radiative transfer model (RTM). The final tropospheric NO_2_ VCD can be derived after subtracting the stratospheric contribution and AMF conversion:

2 $$V_{\mathrm{tropo}} = \frac{{S - V_{\mathrm{strat}} \times M_{\mathrm{strat}}}}{{M_{\mathrm{tropo}}}} = \left(\frac{S}{{M_{\mathrm{strat}}}} - V_{\mathrm{strat}}\right) \times \frac{{M_{\mathrm{strat}}}}{{M_{\mathrm{tropo}}}}$$

where *V*_tropo_ and *V*_strat_ denote tropospheric and stratospheric *V*, respectively. *M*_tropo_ and *M*_strat_ represent tropospheric and stratospheric *M*, respectively. Details of the stratospheric estimation and AMF calculation are provided in the “Materials and methods” section.

**Fig. 2:**
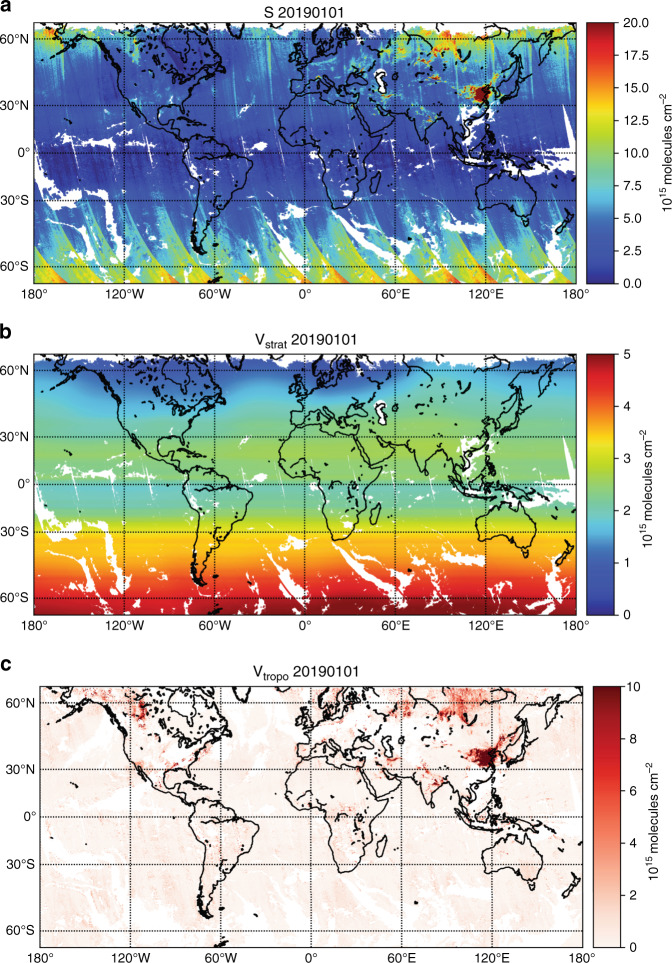
An example of EMI NO_2_ retrieval on 1 January 2019. The total SCDs (S), stratospheric VCDs (*V*_strat_), and tropospheric VCDs (*V*_tropo_) retrieval of NO_2_ are shown in **a**, **b**, and **c**, respectively. Note that satellite ground pixels affected by clouds are indicated in white

Figure 2 shows an example of EMI NO_2_ retrieval of S, *V*_strat_, and *V*_tropo_ on 1 January 2019. Enhanced NO_2_ levels are observed in Eastern China, India, and the Middle East.

### Algorithm improvements

A prototype EMI NO_2_ retrieval is developed before launch. The prototype algorithm is very similar to the operational OMI NO_2_ retrieval^17^. However, due to unexpected issues, i.e., low signal-to-noise ratio at the edges of the spectral channels, bad irradiance measurement due to a diffuser calibration issue, and spectral saturation issue, the NO_2_ retrieval setting must be further optimized to address these issues. A series of sensitivity tests, including cloud correction, fitting wavelength range, reference selection, and spectral precalibration, have been performed to optimize the settings for tropospheric NO_2_ VCD retrieval. Table 1 lists the updated retrieval settings of the EMI NO_2_ retrieval. Parameters used in the OMI QA4ECV NO_2_ retrieval^17^ are also listed for reference.

**Table 1:** NO_2_ retrieval settings used in the EMI and OMI NO_2_ algorithms

Configurations and parameters	OMI NO_2_ product (Boersma et al.^17^)	EMI NO_2_ product (in this study)
NO_2_ SCDs fitting		
Wavelength range	405–465 nm	420–470 nm
Radiometric calibration	Using calibrated (ir)radiance^30^	Recalibrated the earth radiance measurements
Reference spectrum	Solar irradiance averaged between 2005−2009 (ref. ^30^)	Daily earth radiance over the remote Pacific
Instrument slit function	Preflight measured^31^	Calibrated online by using solar atlas^22^, Gaussian shape assumed.
NO_2_ AMF calculations		
Radiative transfer model (RTM)	Doubling-Adding KNMI (DAK) model^17^	Vector Linearized Discrete Ordinate Radiative Transfer (VLIDORT) model^14^
Calculation method	Lookup table interpolation	Lookup table interpolation
A priori NO_2_ profile	The global chemistry Transport Model version 5 (TM5)^32^ simulations at 1 × 1°	The GEOS-Chem v10-01 at 2 × 2.5° for the global domain^33^, and WRF-Chem v3.7 at ~20 km for the China domain^34^
Stratospheric–tropospheric separation	Data assimilation^17^	Reference sector method^14^

**Fig. 3:**
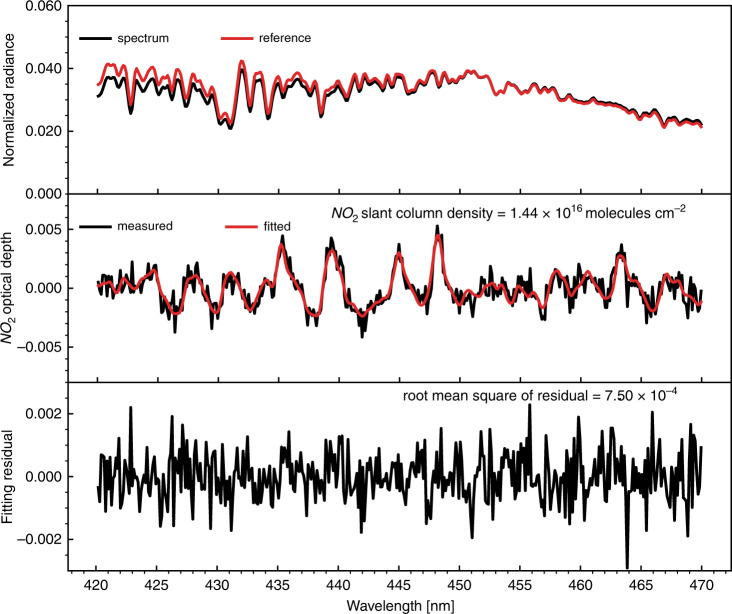
An example of NO_2_ SCD retrieval from the EMI measurement spectra over Beijing on 15 May 2019. The top panel shows the fitted spectra and reference. The middle panel shows the measured and fitted NO_2_ optical depth. The spectral fit residual is shown in the bottom panel

The EMI NO_2_ fitting range is shifted slightly from 405–465 nm (OMI operational NO_2_ setting^17^) to 420–470 nm to avoid the lower signal-to-noise ratio region at the edges of the VIS1 channel^12^. Figure 3 illustrates an example of the retrieval of NO_2_ SCD, i.e., the NO_2_ amount integrated along the optical path in the atmosphere, by applying the DOAS fit to the EMI-measured spectrum.

The spectral saturation issue (i.e., the analogue photon signal reaches the maximum digital value of the CCD detector) is critical for EMI observations over bright clouds due to its high surface reflectance. Supplementary Figure 1 shows the global spatial pattern of the root mean square (RMS) of the spectral fitting residual, cloud radiance fraction from TROPOMI observations, and the true color image from the MODIS-Aqua instrument on 1 January 2019. The spatial pattern of the fitting residual RMS is correlated to the cloud pattern. Therefore, we filtered pixels with relatively large spectral fitting residuals, i.e., the RMS values >0.004.

The key calibration data measured during the on-ground calibration^12^ seem unsuitable for EMI on-orbit measurements due to the degradation and stability of the instrument in the complex space environment (e.g., cosmic radiation exposure^18^ and possible instrument changes since launch^19^). Therefore, we recalibrated the EMI earth radiance measurements by comparing the EMI radiance to TROPOMI measurements and RTM simulations. An advantage of DOAS is that it does not rely on precisely calibrated radiance and is less sensitive to the variability in radiometric calibration than other methods based on discrete radiance (e.g., SBUV and TOMS ozone retrieval algorithms^20^).

**Fig. 4:**
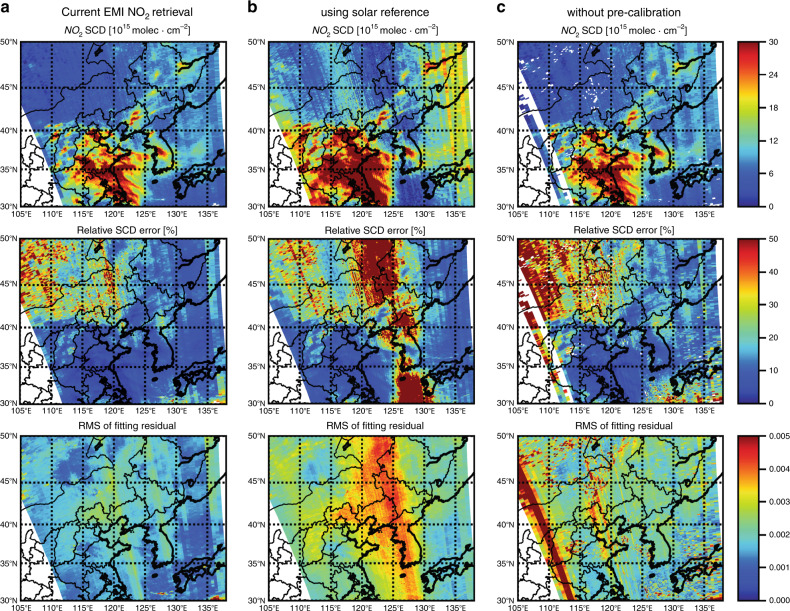
Comparisons of NO_2_ spectral retrieval results from orbit 03241 on 17 December 2018. **a** The current EMI NO_2_ retrieval in Table 1. **b** same as in **a** but using solar reference. **c** same as in **a**, but without spectral precalibration. The resulting NO_2_ SCD, relative uncertainty, and RMS of the fitting residual are shown in the upper, middle, and bottom panels, respectively. The fitted NO_2_ SCD and its uncertainty are masked in the white color when RMS > 0.004

Figure 4 shows the comparisons of NO_2_ SCDs for one orbit on 4 January 2019 retrieved using these spectral fitting scenarios: (a) current settings of the EMI NO_2_ retrieval listed in Table 1; (b) using the measured irradiance spectrum as a reference; and (c) same as in (a) but without spectral precalibration. Irradiance spectra measured by EMI are currently accounting for some calibration issues, and these issues are probably related to the interference of the space environment on the hemispheric reflectance of solar diffusers^18^. NO_2_ SCDs retrieved with irradiance as a reference show large biases and errors, particularly the central part of the measurement swath (Fig. 4b). Therefore, it is not optimal to use the direct sun irradiance spectra as a reference. To avoid the influence of abnormal irradiance spectra, we use cloud-free earth radiance measurements over the Pacific Ocean as a reference^21^. Compared to using solar irradiance as a reference, using earth radiance as a reference greatly reduced the spectral noise in the fit residual (Fig. 4a, b), which is likely related to the differences between spectra measured with the solar and earth-viewing modes^21^. The mean RMS of fitting residual by using earth radiance as a reference over cloud-free regions is 30% smaller than that with irradiance as a reference, as shown in Fig. 4a, b.

Although using radiance as a reference improved the spectral retrieval, the radiance reference also contains a NO_2_ absorption signal. Therefore, we must calculate the SCD offset to compensate for the residual NO_2_ signal in the reference spectrum. The SCD offset is calculated using the NO_2_ AMFs multiplied by the a priori NO_2_ profile taken from the GEOS-Chem model simulations (Supplementary Fig. 2). The NO_2_ simulation over clean remote regions is generally consistent with independent satellite observations, with a monthly mean bias of <0.26 × 10^15^ molecules cm^−2^ (Supplementary Fig. 3). Then, the SCD offset is added back to the NO_2_ SCDs. Note that the reference spectra are selected for each cross-track row to minimize the cross-track bias due to instrument artifacts. The systematic cross-track bias in EMI NO_2_ SCDs (the so-called “stripes”, see Supplementary Fig. 4) is also observed for the OMI and TROPOMI products, and this bias can also be mitigated by using earth radiance as the reference spectrum^21^.

To account for the small variation in the spectral alignment due to the thermal variation in space^12^, we calibrated the additional spectral shift or squeeze and instrument slit function through cross-correlation with a high-resolution solar spectrum atlas^22^ prior to the NO_2_ DOAS fitting. The precalibrated measurement spectra lead to an ~30% smaller SCD fitting uncertainty than using initial calibration parameters (Fig. 4c), as well as a fit residual, and the SCD is nearly unchanged (within ~3.3%).

## Discussion

**Fig. 5:**
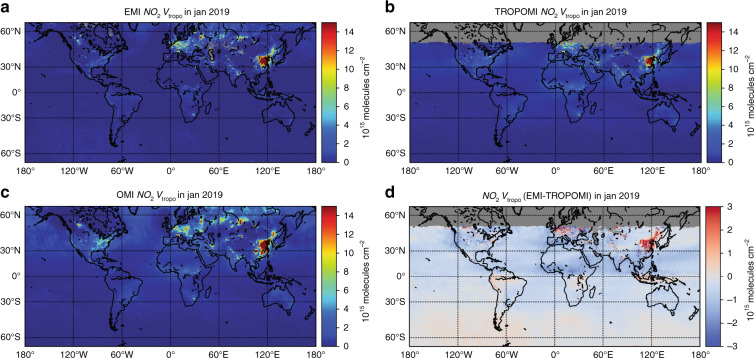
Validations of EMI tropospheric NO_2_ VCDs against OMI and TROPOMI observations. The global distribution of tropospheric NO_2_ VCD in January 2019 is shown for **a** EMI, **b** TROPOMI, and **c** OMI. **d** shows the difference between EMI NO_2_ in **a** and TROPOMI NO_2_ in **b**

**Fig. 6:**
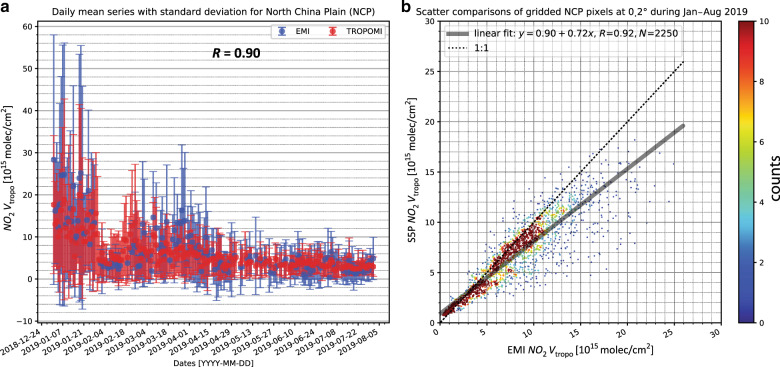
Comparisons between tropospheric NO_2_ VCDs from EMI and TROPOMI. **a** Shows the daily mean time series of tropospheric NO_2_ VCD over the North China Plain (NCP) from EMI and TROPOMI. Error bars indicate the 1σ standard deviation. **b** The density scatter plot of NO_2_ VCDs over the NCP. Data are regridded to a resolution of 0.2° and averaged for the period of January–August 2019

The tropospheric NO_2_ VCDs retrieved from EMI spectra are first validated against the OMI QA4ECV NO_2_ products and the operational TROPOMI NO_2_ products^23^. EMI measurements are compared to the OMI and TROPOMI products due to their similar instrument characteristics, i.e., the push-broom design, spectral bands, and near-noon overpass time at ~13:30. Note that the TROPOMI NO_2_ product generally followed the OMI QA4ECV NO_2_ retrieval algorithm, but TROPOMI has a higher signal-to-noise ratio and spatial resolution^23^. Figure 5 shows the monthly averaged NO_2_ VCDs measured by EMI, OMI, and TROPOMI in January 2019. EMI NO_2_ VCDs generally show similar spatial patterns and amplitudes of NO_2_ VCDs compared to OMI and TROPOMI, while finer-scale details of NO_2_ are captured by the satellite instrument with a higher spatial resolution. The EMI dataset overestimates NO_2_ VCDs by up to 50% over polluted regions, such as the North China Plain (NCP) and India (Fig. 5d) compared to the TROPOMI observations. The spatiotemporal correlations between EMI NO_2_ and TROPOMI NO_2_ were also evaluated. For data taken from January to August 2019, the correlation coefficient (*R*) of daily mean NO_2_ VCD time series over NCP between EMI and TROPOMI is 0.90, while the spatial correlation coefficient (*R*) of mean NO_2_ VCDs over the NCP is 0.92 (Fig. 6). The remaining discrepancies between EMI and TROPOMI are mainly due to the NO_2_ vertical profile used in the tropospheric AMF calculation, while the spectral fitting method (<3%) and stratospheric estimation method (<10%) only show a minor contribution (Supplementary Fig. 5).

**Fig. 7:**
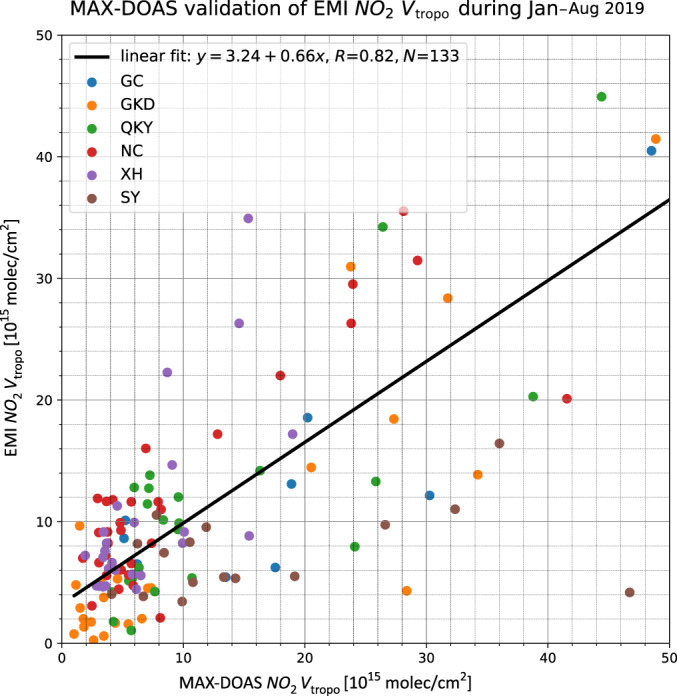
Scatter plot of EMI tropospheric NO_2_ VCD against ground-based MAX-DOAS observations in northern China. Measurements from six different measurement sites are used in the comparison: the Gucheng site (GC, 39.149°N, 115.734°E) in the Hebei Province, the Guokeda site (GKD, 40.408°N, 116.675°E) in Beijing, the Qikeyuan (QKY, 39.9472°N, 116.3206°E) site in Beijing, the Nancheng (NC, 39.781°N, 116.127°E) site in Beijing, the Xianghe site (XH, 39.750°N, 116.095°E) in the Hebei Province, and the Shengyang site (SY, 41.812°N, 123.401°E) in the Liaoning Province

The EMI tropospheric NO_2_ VCDs are also compared to the ground-based NO_2_ measurements from the Multi-AXis Differential Optical Absorption Spectroscopy (MAX-DOAS) instruments over northern China. A good agreement with a Pearson correlation coefficient (*R*) of 0.82 is found between the two datasets during January–August 2019 (Fig. 7, Supplementary Fig. 6). However, EMI generally underestimates tropospheric NO_2_ VCDs by 30% compared to MAX-DOAS. The biases can be explained by the difference in spatial coverage between the ground-based and satellite observations^24,25^. In general, both satellite and ground-based validations of EMI NO_2_ measurements show good agreement with correlation coefficients (*R*) of 0.8–0.9, indicating that a new EMI tropospheric NO_2_ retrieval provides reliable results for the investigation of air pollution distribution.

## Materials and methods

### The stratosphere–troposphere separation

The stratospheric contribution of NO_2_ must be subtracted from the total NO_2_ column to derive the tropospheric NO_2_ column. In the EMI NO_2_ retrieval, we used the STRatospheric Estimation Algorithm from Mainz^14^ to estimate the stratospheric contribution, which is based on the assumption that there is negligible contribution of tropospheric NO_2_ columns over the remote Pacific and cloudy pixels in the middle latitudes. The weighting factors based on cloud and polluted regions, which determines their impacts on the stratospheric estimate, are assigned to each satellite pixel. Subsequently, spatial smoothing based on weighted convolution is used to estimate the global stratospheric column.

### NO_2_ AMF calculations

The EMI NO_2_ AMFs of each atmospheric layer (i.e., Box-AMFs) are calculated at 445 nm by the linearized pseudospherical vector model VLIDORT^26^ version 2.7. In addition to the solar and satellite-viewing geometries provided in the level 1 data, additional atmospheric and surface information are needed in the AMF calculations. Surface albedo at 442 nm is taken from the OMI minimum earth’s surface Lambertian equivalent reflectance^27^ and interpolated to the EMI footprints. Considering the same local overpass time between EMI and TROPOMI, cloud top pressure and cloud fraction from TROPOMI^28^ are used for the calculations of EMI NO_2_ AMFs. A priori NO_2_ profiles are taken from the high-resolution (~20 km) WRF-Chem simulations for the China domain and from GEOS-Chem simulations at the resolution of 2 × 2.5° for the global domain (Supplementary Fig. 7). The spatial resolution of the NO_2_ a priori profile is reportedly one of the dominant uncertainty sources during the NO_2_ AMF calculations^29^. To expedite the calculation, these box-AMFs are precalculated and stored in the six-dimensional lookup table. Then, the box-AMF for each EMI observation can be derived by interpolating within the lookup table.

## Supplementary information

Supplementary Information

## Data Availability

The EMI level 1 and NO_2_ datasets are available from Cheng Liu (chliu81@ustc.edu.cn) upon reasonable request. The OMI QA4ECV NO_2_ and TROPOMI NO_2_ datasets are available from http://www.temis.nl/airpollution/no2.html.
