# Supplementary material for: First observation of tropospheric nitrogen dioxide from the Environmental Trace Gases Monitoring Instrument onboard the GaoFen-5 satellite
Source: Light Sci Appl. 2020 Apr 20;9:66. doi: 10.1038/s41377-020-0306-z (PMC7170962; doi:10.1038/s41377-020-0306-z)
Supplement: Supplementary file 1 — Supplementary Information [file 41377_2020_306_MOESM1_ESM.pdf]

Supplementary Information for

First observation of tropospheric nitrogen dioxide from the Environmental trace gases Monitoring Instrument onboard the GaoFen-5 satellite

Chengxin Zhang[1,#], Cheng Liu[4,2,3,6,#,*], Ka Lok Chan[5,*], Qihou Hu[2], Haoran Liu[1], Bo Li[1], Chengzhi Xing[1], Wei Tan[2], Haijin Zhou[2], Fuqi Si[2], Jianguo Liu[2,3]

[1]School of Earth and Space Sciences, University of Science and Technology of China, Hefei, 230026, China
[2]Key Laboratory of Environmental Optics and Technology, Anhui Institute of Optics and Fine Mechanics, Chinese Academy of Sciences, Hefei, 230031, China
[3]Center for Excellence in Regional Atmospheric Environment, Institute of Urban Environment, Chinese Academy of Sciences, Xiamen, 361021, China
[4]Department of Precision Machinery and Precision Instrumentation, University of Science and Technology of China, Hefei, 230026, China
[5]Remote Sensing Technology Institute (IMF), German Aerospace Center (DLR), Oberpfaffenhofen, Germany
[6]Key Laboratory of Precision Scientific Instrumentation of Anhui Higher Education Institutes, University of Science and Technology of China, Hefei, 230026, China

[#]These authors contributed equally to this work

[*]Corresponding authors: Cheng Liu (chliu81@ustc.edu.cn), Ka Lok Chan (ka.chan@dlr.de)

Supplementary Figures

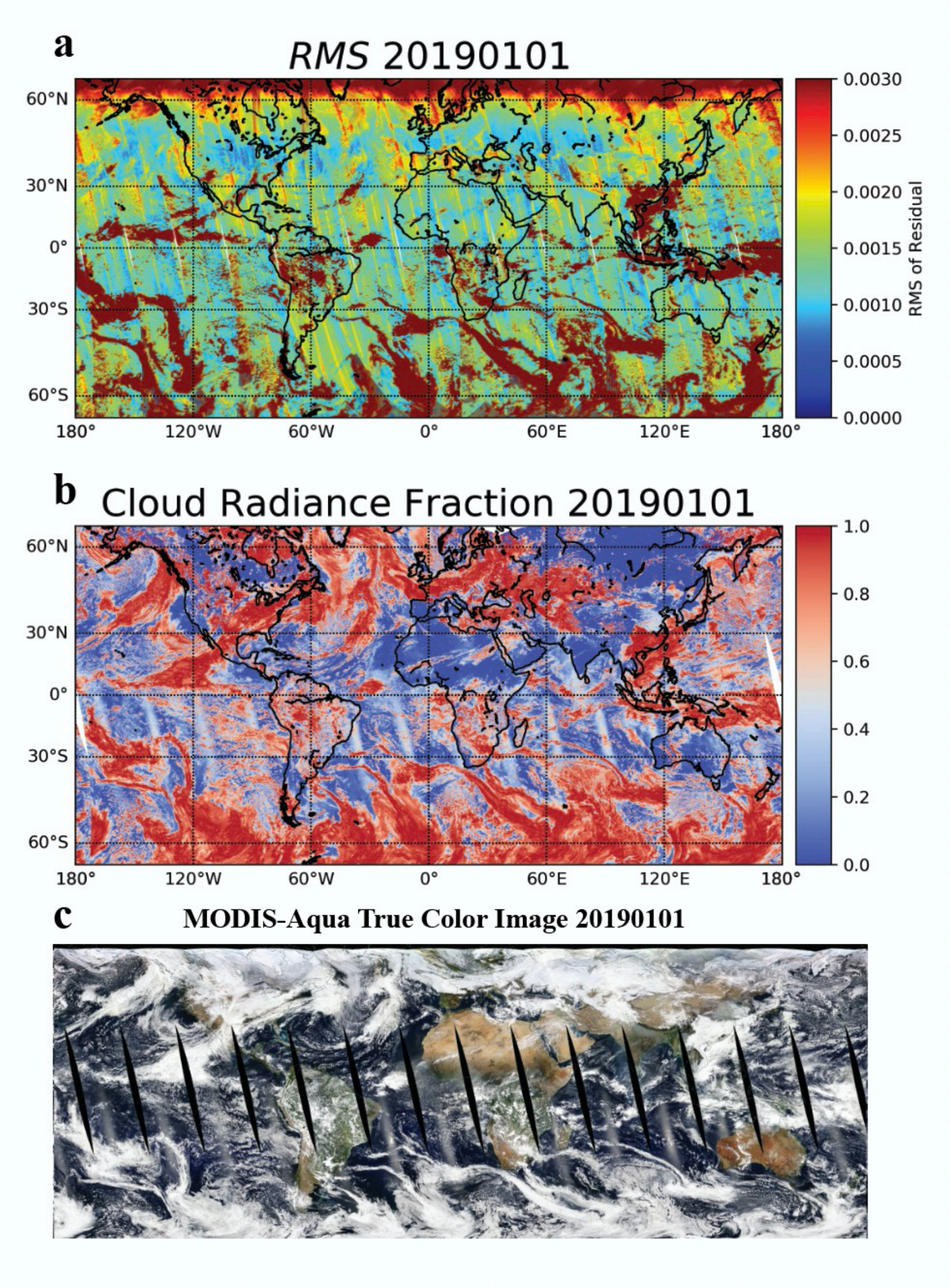

Figure S1. The root mean square of the spectra fitting residual during EMI NO_2 retrieval, cloud radiance fraction from TROPOMI cloud products, and corrected reflectance true color image by MODIS-Aqua on 1 January 2019 were shown in (**a**), (**b**), (**c**), respectively.

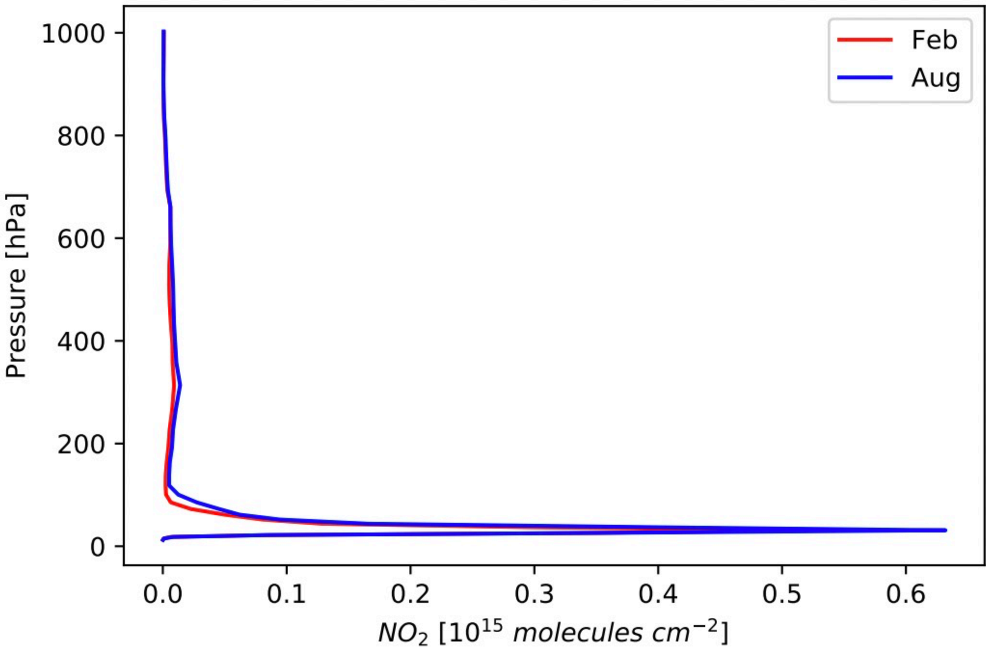

Figure S2. Monthly mean NO_2 profiles simulated by the GEOS-Chem at 15°N, 160°E, remote pacific for February and August 2019.

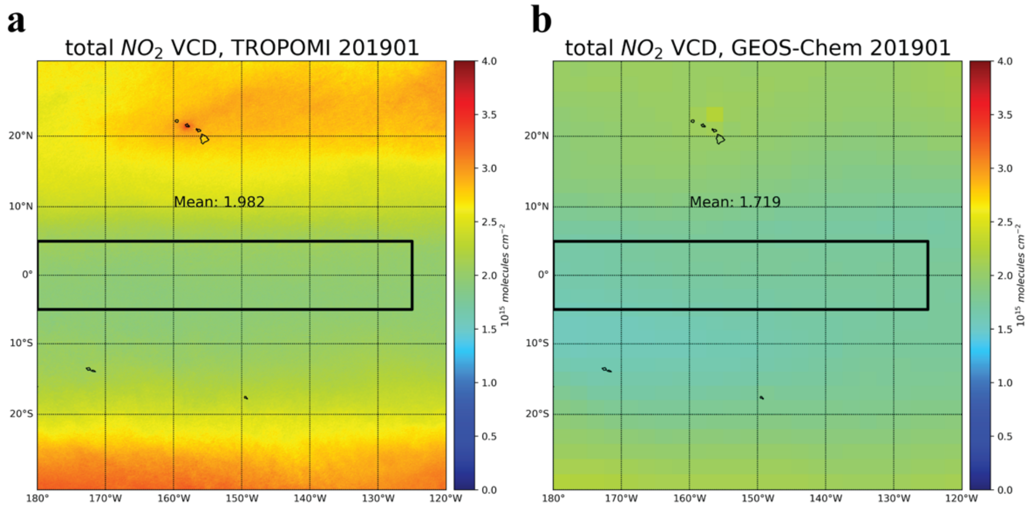

Figure S3. The total NO_2 VCD averaged during January 2019 from **(a)** TROPOMI observations and **(b)** GEOS-Chem simulations. The black rectangle denotes the reference sector region for earthshine reference selection. And the corresponding mean value of NO_2 VCD over this region are noted in text.

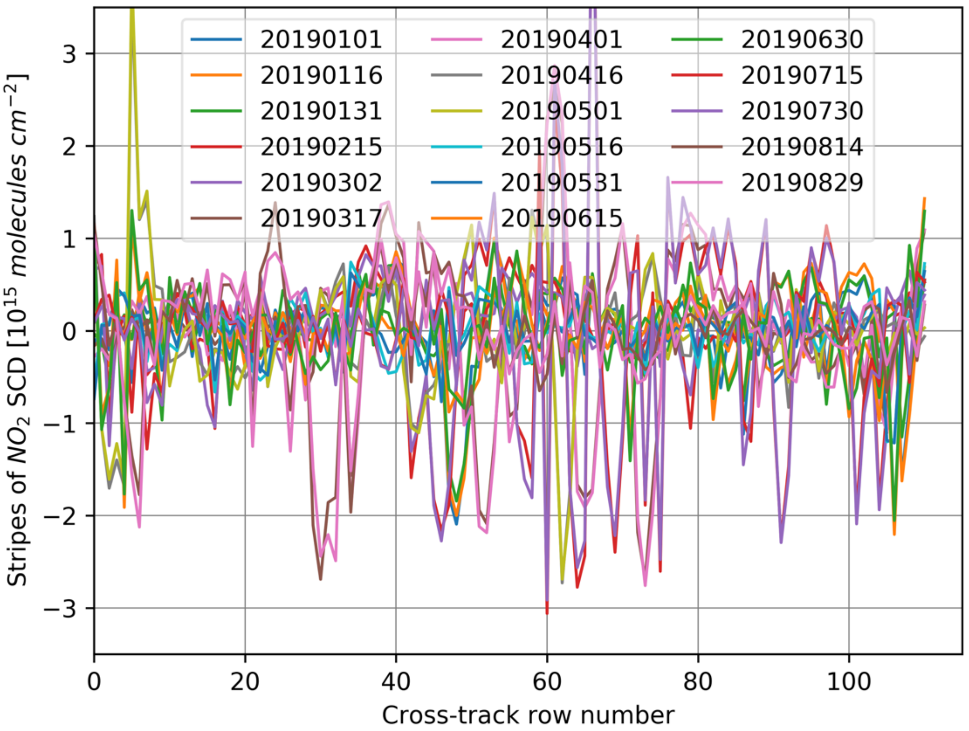

Figure S4. The cross-track bias, i.e., stripes, of EMI NO_2 SCDs. The stripe patterns for different day are shown with different color.

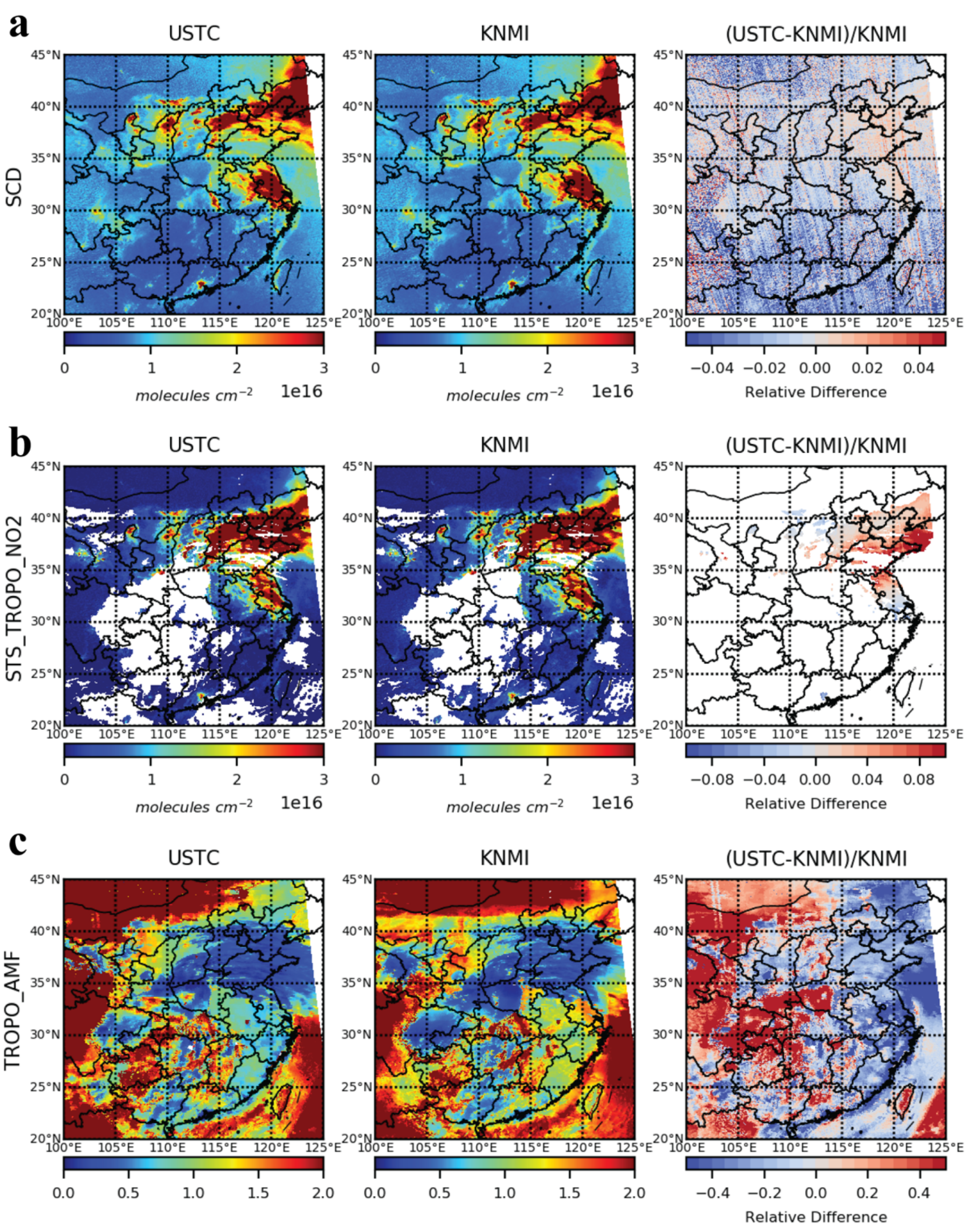

Figure S5. Comparisons of NO_2 SCDs retrieval (**a**), tropospheric NO_2 retrieval using different stratospheric-tropospheric separation (STS) methods (**b**), and tropospheric NO_2 AMFs (**c**) between the EMI NO_2 retrieval algorithm and the operational TROPOMI NO_2 product for TROPOMI measurement on 18 January, 2019. Results from EMI NO_2 algorithm are labeled as USTC (in left panel), while the operational TROPOMI NO_2 are labeled as KNMI (in central panel). The relative differences between two are shown in the right panel. Note that for STS method, USTC algorithm also used AMF and SCD results from the operational NO_2 datasets. Cloudy scenes and unpolluted regions (i.e., cloud radiance fraction larger than 0.5 and tropospheric NO_2 column greater than 2.0×10^{16} molecules cm^{-2}) are masked by white in (**b**).

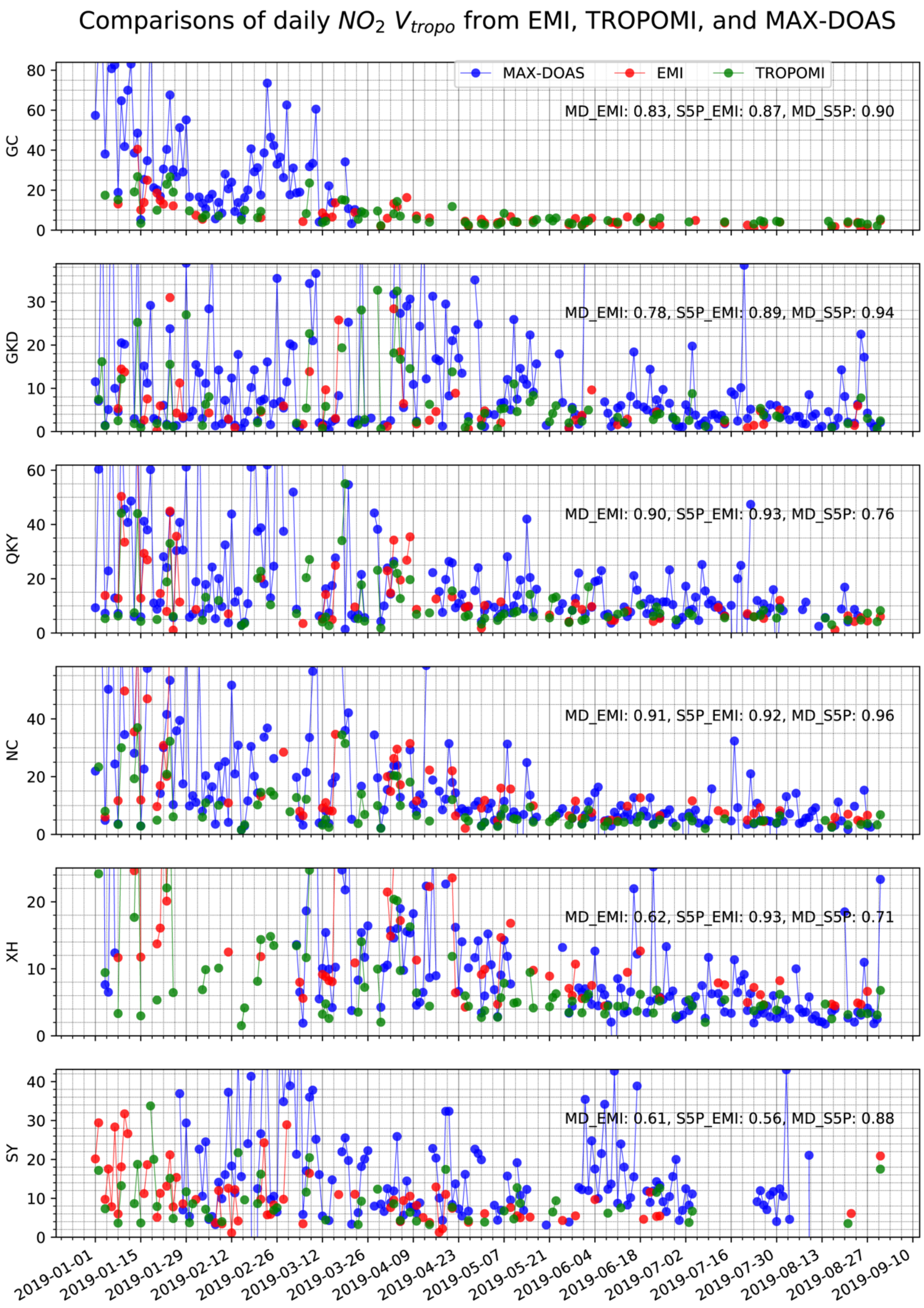

Figure S6. Similar to Fig. 7, but for daily series comparisons between tropospheric NO_2 VCD retrievals from the EMI, TROPOMI/S5P, and the MAX-DOAS instruments. The correlation coefficients (R) between either two of these measurements are indicated by the text.

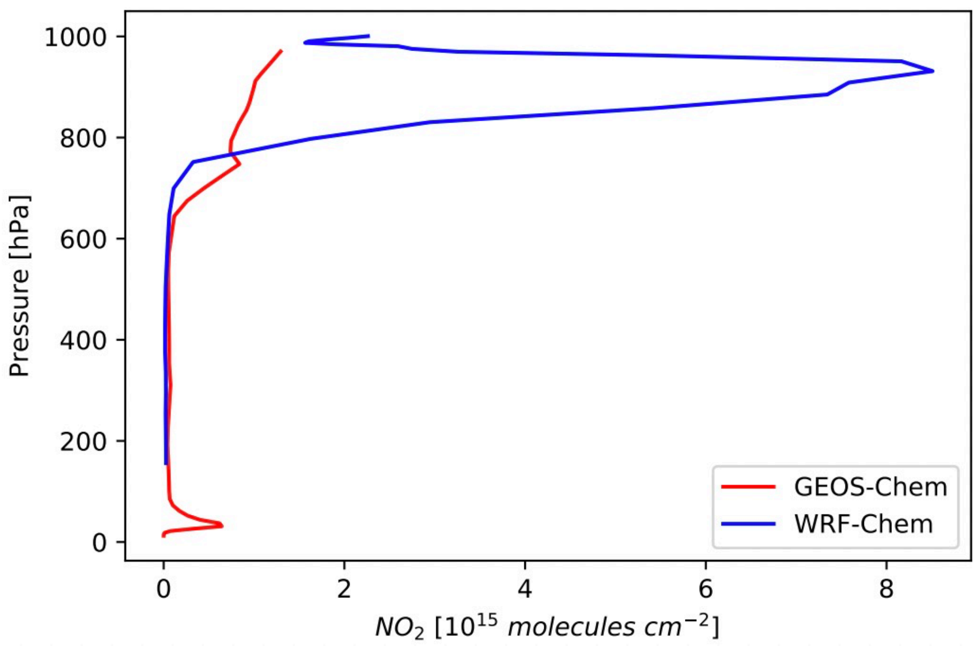

Figure S7. Comparisons of monthly mean NO_2 profiles simulated by the GEOS-Chem and WRF-Chem models. The location is selected at Beijing center for June 2019.
